# A Long-term Consistent Artificial Intelligence and Remote Sensing-based Soil Moisture Dataset

**DOI:** 10.1038/s41597-023-02053-x

**Published:** 2023-03-22

**Authors:** Olya Skulovich, Pierre Gentine

**Affiliations:** Columbia University, Earth and Environmental Engineering Department, New York, NY 10027 USA

**Keywords:** Hydrology, Climate and Earth system modelling

## Abstract

The Consistent Artificial Intelligence (AI)-based Soil Moisture (CASM) dataset is a global, consistent, and long-term, remote sensing soil moisture (SM) dataset created using machine learning. It is based on the NASA Soil Moisture Active Passive (SMAP) satellite mission SM data and is aimed at extrapolating SMAP-like quality SM back in time using previous satellite microwave platforms. CASM represents SM in the top soil layer, and it is defined on a global 25 km EASE-2 grid and for 2002–2020 with a 3-day temporal resolution. The seasonal cycle is removed for the neural network training to ensure its skill is targeted at predicting SM extremes. CASM comparison to 367 global *in-situ* SM monitoring sites shows a SMAP-like median correlation of 0.66. Additionally, the SM product uncertainty was assessed, and both aleatoric and epistemic uncertainties were estimated and included in the dataset. CASM dataset can be used to study a wide range of hydrological, carbon cycle, and energy processes since only a consistent long-term dataset allows assessing changes in water availability and water stress.

## Background & Summary

Soil moisture (SM) is a key climatic variable^1–7^ essential for a wide range of hydrological, carbon cycle, and energy processes. Indeed, SM influences photosynthesis, soil respiration, evapotranspiration, runoff, surface heat flux partitioning, and the occurrence and intensity of floods and droughts^2,7–12^. Through the direct effect of soil dryness and indirect effect of atmospheric vapor pressure deficit, the seasonal and interannual variability in soil moisture is a key determinant of the land capacity to act as a carbon sink^13–16^. Understanding and monitoring global trends in SM has the potential to shed light on climate change processes and their future^2^.

The growing interest in soil moisture has led to significant efforts devoted to collecting *in-situ* and developing remote soil moisture data. However, to date, a truly coherent and comprehensive soil moisture database with systematic data quality checks does not exist on a global scale. Such a global SM dataset can only be created using remote sensing products. SM remote sensing has advanced tremendously in recent years, however, further back in time, less data is available both spatially and temporally and the quality and consistency degrade substantially^17^. Attempts to tackle this issue have included combining data from several satellites into one dataset from either passive (observes natural thermal emission) or active (compares received to the transmitted microwave pulse) or a combination of active and passive microwave remote sensing platforms.

Significant efforts to merge SM products from different satellite sensors into a single dataset^18–20^ resulted in ESA CCI (European Space Agency Climate Change Initiative) surface soil moisture. More recently, Rodriguez *et al*.^21^ used a neural network (NN) to reproduce data of the Soil Moisture and Ocean Salinity (SMOS) SM, with a data quality similar to the original SMOS SM. Another example of soil moisture retrievals from both active and passive microwave observations^22^ utilized a different methodology (NN) and targeted a modeled SM product instead of retrieval for the training. Another example of microwave observations merging from multiple sensors with NN was presented in a very beginning of SMAP mission^23^. Others^24–26^ used statistical methods (e.g. triple collocation, least squares weighting, unweighted averaging, emergent constraints, etc.) to blend the different products, satellite observations, reanalysis, or offline land surface model simulation. Still, merging various sensor retrievals *a posteriori* requires major assumptions on the relationship between those sensors and on their distribution, which can nonetheless vary both spatially and temporally, and this relationship is likely nonlinear and state-dependent. As opposed to empirical matching approaches, an NN has the advantage of being both nonlinear, and state-dependent, and thus naturally imposing a global distribution matching. NN creates a data set that is directly consistent with the target data (either an SM model or an SM retrieval from other sources) and does not need any *posteriori* distribution or bias correction, as it is directly handled by the neural network.

L-Band is considered for topsoil SM retrievals^27^. Currently, both SMOS and SMAP are operating L-band satellite missions, however, SMOS retrievals are generally affected by radio frequency interference (RFI), whereas SMAP mission satellite is equipped with RFI mitigation hardware. The main limitation of the SMAP SM product is that it is only available from March 31, 2015, when the mission was launched, and hence does not have enough temporal coverage to adequately asses interannual and decadal SM variability. In this work, a high-quality consistent long-term remote sensing SM dataset is created based on SMAP data as the target. The emphasis of the product is its consistency in matching SMAP SM to avoid the emergence of artificial trends in the data due to inaccurate distribution matching for data from different sources. Specifically, a NN is trained to reproduce SMAP SM from SMOS, AMSR-E, and AMSR2 brightness temperatures (TBs). To achieve this, two major strategies are utilized. First, since the seasonal cycle comprises the majority of the SM and TB signals that may compromise the NN ability to learn the actual SM variability, the seasonal cycle was removed, and the NN was trained on the residuals TB and SM. Second, a multi-stage NN training strategy with transfer learning was used to achieve a smooth satellite to satellite transition. The resulting global product has a temporal resolution of 3 days and a spatial grid resolution of 25 km. It shows R^2^ = 0.97 to the original SMAP for 2015–2020, and median correlation 0.66 when compared to 367 sites with *in-situ* SM measurements for 2002–2020. Additionally, aleatoric and epistemic uncertainty estimates are provided in order to give uncertainty quantification of the retrieval as a function of time and location.

## Methods

**Fig. 1:**
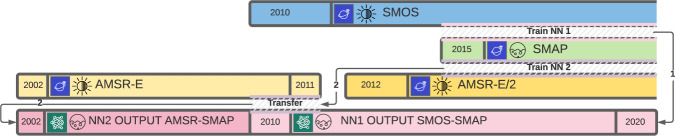
NN training scheme. Each satellite mission is color-coded to facilitate visibility: SMOS is blue, SMAP is light green, AMSR is yellow, and the NN output product is pink. Brightness temperature data is depicted as a sun icon, soil moisture data is depicted as a circle icon. Satellite data is depicted as a navy blue icon with a planet and an orbiting satellite; NN product is depicted as a dark green icon with a schematic of a NN. Gray shaded areas represent data overlap that was used for NN training, including transfer learning.

The methodological section of this study is organized as follows. First, all used datasets are briefly described and data handling strategies are outlined. This includes justification of data averaging to a 3-day resolution (Section Data handling). Then, one of the most important features of the study - data deseasonalizing and motivation behind it - is described in Section Seasonal cycle. Next, some alternative approaches, that were investigated by the authors but showed sub-optimal performance and were not utilized in the final dataset-building procedure are mentioned in Section Preliminary investigation. The core of the dataset-building procedure is described in Section Training Scheme and Fig. 1. Finally, the methodological approach to assessing the uncertainty of the CASM dataset is provided in Section Uncertainty.

### Datasets

To create our Consistent AI-based Soil Moisture (CASM) dataset^28,29^, soil moisture from the NASA SMAP mission was used as a target with brightness temperatures from SMOS, AMSR-E, and AMSR2 as input. The final product was compared to *in-situ* measurements collected through International Soil Moisture Network.

#### SMAP

NASA’s Soil Moisture Active Passive (SMAP) mission uses an L-band radiometer to retrieve surface soil moisture. The measurements are sensitive to water content in the top 5 cm of soil^30^. SMAP real-aperture radar footprint resolution is 29 km by 35 km. In this study, the enhanced passive microwave 25-km Equal-Area Scalable Earth Grid 2.0 (EASE2) global daily product from descending SMAP orbit (6:00 AM, local solar time) was used since morning retrieval of SM is less impacted by vertical heterogeneity in the land-surface temperature than the a more adequately indicates the actual SM^31^. The soil moisture data (in m^3^/m^3^) is taken from March 31, 2015 until August 27, 2020. Soil moisture SMAP products are publicly available at the National Snow and Ice Data Center, however, the 25-km grid data is currently depreciated. The 25-km grid data was chosen to correspond to the other remote sensing SM data sources available.

#### SMOS

Gridded to the matching 25-km EASE2 projection, SMOS data from^32^, in particular, the L3TB global daily product that includes all brightness temperatures from the SMOS satellite is used. The SMOS brightness temperature (in degrees K) includes 14 incidence angles (from 2.5° to 62.5°) in H- and V-polarization is taken from January 12, 2010, until August 27, 2020. SMOS instrumental spatial resolution is 35–50 km. Only ascending (6 a.m. local time) orbit is used to minimize the impact of time of day on canopy temperature.

#### AMSR-E and AMSR2

The Advanced Microwave Scanning Radiometer - Earth Observing System (AMSR-E)^33^ is a NASA EOS Aqua satellite instrument. AMSR-E Equal-Area Scalable Earth (EASE-Grid, not equivalent to EASE2) gridded brightness temperature data provides global passive microwave measurements at 25 km resolution. The data is available from six frequency channels: 6.9 GHz, 10.7 GHz, 18.7 GHz, 23.8 GHz, 36.5 GHz, and 89.0 GHz in H- and V- polarization each and has temporal coverage from June 19, 2002, to September 27, 2011.

The AMSR2 data is taken from The AMSR-E/AMSR2 Unified Level-2B land product^34^. For this study, the period that temporarily overlaps with SMAP data was used. The brightness temperatures are provided in the 25 km EASE-Grid (EASE and EASE2 grids are not directly compatible). All of the available 6.9 GHz, 10.7 GHz, 18.7 GHz, 23.8 GHz, 36.5 GHz, and 89.0 GHz H-polarized and V-polarized brightness temperatures (12 in total) were used for AMSR-E and AMSR2.

#### In-situ data

The International Soil Moisture Network (ISMN)^35–37^ comprises standardized SM observations from around the globe. The *in-situ* measurements collected in the ISMN are measured using various types of sensors, however, the collection is quality controlled (yet the quality varies drastically as will be discussed) and is widely used in literature as a reference for SM products evaluation. The full collection was filtered as outlined below. In the ISMN dataset, all stations that measured SM in the top 5 cm of the soil at some point between 2002 and 2020 (the temporal span of our dataset) were considered. Among those, the stations that had less than 90% of the data with the good quality flag were excluded. The whole station was excluded in that case rather than just datapoints with the “bad” quality flag only since often consistent bad data indicated a large bias or unrealistic variations in the measurements. For the remaining stations, a 3-day mean SM was used for smoothing and better comparison to our CASM product. Then, only the stations that had at least 100 observations were used. Pearson correlation, root mean square error (RMSE), and unbiased RMSE between the station data and the closest CASM grid point SM data were calculated as a measure of correspondence between the *in-situ* and observational datasets. The following subnetworks’ data were included for the comparison: ARM, FLUXNET-AMERIFLUX, PBO-H2O, SCAN, SOILSCAPE, USCRN, USDA-ARS, AMMA-CATCH, DAHRA, FR-Aqui, HOBE, HYDROL-NET Perugia, MySMNet, ORACLE, OZNET, REMEDHUS, SASMAS, SMOSMANIA, TAHMO, TERENO, VAS, and WSMN, for references, see Supplementary Table S1.

#### Auxiliary data

Static soil type data and leaf area index (LAI) data were tried as auxiliary inputs during the NN preliminary study. Soil type data, based on a machine learning global prediction of basic soil properties^38^, was used as a static variable in the NN input configurations. A long-term MODIS LAI product^39^ was tried as a dynamic auxiliary input during the NN preliminary study. Including more auxiliary datasets can potentially improve SM retrievals, however, it is also a source of additional challenges due to the limited spatial and temporal data availability, the introduction of additional biases when using patched data from multiple sources, and the additional complication of the model. In NN exploration (Section Preliminary investigation), it was decided against using these auxiliary datasets, as they did not improve the predictions of the final SM product.

### Data handling

All datasets were re-gridded to match SMOS grid using *gdal* bilinear resampling method^40^. Next, the data was temporarily averaged to 3-day means. This step is necessary to have consistent input resolution for the NN training needs. When the satellites pass above the Earth, the data they collect in daily swaths do not necessarily cover the entire Earth’s surface (Supplementary Fig. S1A, C). Only the regions that have overlapping data from multiple sources for a particular day were thus considered in the training dataset. We note that some regions may never be sampled in such training data due to opposite satellite orbits (Supplementary Fig. S1A, C). This results in a situation where while the NN results show high correlation *R* and low mean square error (*mse*) on the training and testing sets, these training and testing datasets poorly represent the original data and thus, are unsuitable for generalization and extrapolation beyond the observed time period. For illustration, see Supplementary Fig. S2. Here panel A shows SMAP SM data in the training dataset, i.e. only for the date-location where SMOS data also exists. If we now train an NN with only these data available for training, such NN can achieve high accuracy *R* = *0.93*, *mse* = *0.0015* as illustrated in Supplementary Fig. S2B, Looking at these numerical metrics, one can assume sufficient results are achieved, and NN output SM is a good match for SMAP SM. However, the actual SMAP SM distribution is very different, see Supplementary Fig. S2C). For comparison, the averaged data, i.e. one such 3-day mean block for SMOS TB and SMAP SM is shown in Supplementary Fig. S1B, D. In this case, there is enough overlapping data for successful NN training since now the training data coverage is consistent with the target SMAP coverage. In addition to that, deseasonalization, described below, will reduce signal-to-noise ratio in the data. With that, temporal averaging assist in restoring this ratio by partially smoothing the noise.

### Seasonal cycle

Both brightness temperature and soil moisture demonstrate a strong seasonal cycle over most of the Earth’s surface. Training an NN on data including this strong seasonal cycle might be taken with caution since good network performance may come mainly from fitting the seasonal cycle rather than from the ability to capture extreme conditions. More to it, in SM research, extremes are of utmost importance both for studying extreme conditions like floods and droughts and for studying the climate change-related variability of these events. To mitigate this seasonal cycle issue, for all datasets and all predictors (brightness temperatures) and response (SM) variables, for every grid point, two new time series were generated: 1) a technical seasonal cycle–a periodic signal with a period of one year, and 2) the residual between the actual data and this periodic signal (i.e., producing anomaly). The algorithm to calculate the seasonal cycle was chosen such that the resulting seasonal cycle is invariant from year to year. Indeed, based on the NN training arguments above, we want this technical seasonal cycle to be a stable periodic signal, constant from year to year with all the deviations from it, that include potential trends, any periodic signals with periods other than one year, and noise, to be in the residual component. The approach is also visualized in Eq. 1: full SM signal is a sum of fixed a seasonal cycle (imposed) and a residual that includes sub-seasonal periodic signals, trends, anomalies, and noise (targeted by the NN).

1 $$\hat{SM}=\mathop{\underbrace{S{M}_{fixed{\rm{\_}}seas{\rm{\_}}cycle}(lat,lon)}}\limits_{imposed}+\mathop{\underbrace{S{M}_{residuals}(lat,lon,TB)}}\limits_{targeted\,by\,NN,includes\,sub-seasonal\,periodic\,signal,\,trends,\,extremes,\,noise}$$

1. Technical seasonal cycle
  Calculating a consistent seasonal cycle for every grid point for remote sensing data is complicated by the fact that the measurements are sparse in time and space. Data filling/interpolation was not considered in order to avoid introducing additional biases. Possible options can include calculating day-of-year multiyear mean or median, sliding window smoothing, locally weighted scatterplot smoothing (*lowess()*^41^), and statsmodel seasonal decompose (*seasonal_decompose()*^41^). However, all these methods return time series that are irregular from year to year. Hence, we decided to use a simple but consistent data fit to a sinusoidal function *Asin*(ω*x* + *ϕ*) + *b* with parameters (amplitude *A*, frequency *ω*, phase ϕ, and shift *b*) that were fitted with the Python function *curve_fit()* including some physical constraints on the parameters (e.g. on the period that had to be equal to one year). This function uses non-linear least squares to fit a function to data. To ensure there were no interannual irregularities due to potential missing data or other issues, a median seasonal cycle was taken for every (latitude, longitude, day-of-year) triple, i.e. at every location, the seasonal cycle was ensured to be exactly the same for any year, regardless of the year. The *curve_fit()* function was only applied if there were at least 40 observations available per location. For the smaller number of observations and when *curve_fit()* could not find the parameters, a median value over all available data points was taken as a technical seasonal cycle at this location. In that respect, the absence of a strong periodic signal does not compromise the algorithm since this absence is favorable for the NN training.
2. Residual: Actual Data minus Seasonal Cycle

As soon as a stable, regular seasonal cycle sinewave was found for each grid point, a simple difference between the actual data and the seasonal cycle defined the residual anomalies that were then used as the input and target of the NN training.

Note that the reason for this signal decomposition is to ensure that the NN can skillfully capture and represent extremes. This seasonal cycle cannot be used to study SM seasonality as it does not necessarily fully correspond to the climatological seasonal cycle at a given location, hence, it is labeled as ‘technical’. Rather, it serves the technical goal of improving NN performance in matching the extremes. As long as a trend or an extreme event is picked up in TB, it will be translated into a trend or an extreme event in SM, alongside with the noise.

### Preliminary investigation

For a preliminary investigation of optimal NN architecture and potential cross-correlations in the input data, pairs of SMOS-SMAP data (SMOS TB in 14 incidence angles and 2 polarizations and SMAP SM) were considered. Naturally, the data collected at different incidence angles of SMOS were highly correlated with correlation coefficients ranging from 0.76 to 0.99. The different incidence angles could potentially lead to more skillful retrieval if they have synergistic information. However, the proportion of missing data increases by 40–53% at the smallest and largest angles (2.5 and 62.5°) in comparison to the 42.5 angle. That is due to SMOS acquisition geometry that results in full swath width obtained only for angles 40° to 45°^42^. In addition, the signal from the smallest and the largest angles is noisy. To balance these issues, data from only four incidence angles were taken: 37.5°, 40°, 42.5°, and 47.5°, in H- and V-polarization each. This subset of data is used in the preliminary analysis, our final choice is indicated at the end of this section.

Next, different machine learning strategies were used and evaluated, either including those cross-variations in incidence angle or not:

1. NN architectures
  Convolutional NN. A convolutional NN (CNN) is a NN that is able to identify signal features from multi-channel data (for example, CNNs are used in computer vision applications for image classification). The use of CNN here is inspired by a hypothesis that signal features can be extracted from multi-angles TB that can be meaningful for better SM prediction.
  Dense NN. A dense NN or multilayered perceptron is a NN with several layers of neurons where each neuron from a previous layer is connected to every neuron of the next layer. This NN allows exploiting nonlinear dependencies in the input data to build a highly non-linear match between the input and the output.
  Branched NN. A branched NN is an arbitrary combination of NN layers of different types with inputs going through different “paths” of layers. In our case, information from SMOS TB first passes through a series of Convolutional layers and then joins an additional dense layer branch that has the auxiliary data as input.
2. Using SMOS TB principal components instead of the actual TB data. A principal component analysis or PCA is an orthogonal transformation of the data from the original feature space, where the features might correlate with each other, to a new feature space (called principal components), where they are linearly uncorrelated. Since TB data from different angles are highly correlated, the PCA can bear the potential to improve SM prediction.
3. The influence and benefits of using the auxiliary data.
4. The influence and benefits of using geographical coordinates data.

The goal of the preliminary check is to empirically find an NN architecture and input configuration that are tailored to the creation of a consistent SM dataset (e.g. the input should not be a source of additional biases). NN performance metrics–training and testing *mse* and *R*–are used to assess the different configurations. In other words, we are looking to answer the following questions: (1) “Is using data from multiple incidence angles and auxiliary data improve the results?” (2) “Can we achieve high performance without including geographical coordinates as the NN input?” and (3) “Does the specified input require complex NN architecture?”.

The results achieved for a dense NN with different input configurations are shown in Supplementary Fig. S3. First, let us notice the influence of multiple incidence angles and auxiliary data. Though if taken individually, a NN with data from 4 incidence angles (“4 SMOS TB to SMAP SM”) performs better than a NN with data from 1 incident angle (“1 SMOS TB to SMAP SM”), and even better with the auxiliary LAI and soil type data, the performance improves significantly only if the geographic coordinates data is added. The brightness temperature used in the NN with only two additional variables–LAI (dynamic) and soil type (static)–is not sufficient for building a proper SM retrieval model. This could have been expected since in the actual SM retrieval models, the set of used auxiliary data is significantly richer (land-water-forest-urban-mountain mask, the grid cell average elevation and slope, soil texture information (static), land cover, surface roughness, precipitation, vegetation parameters, and effective soil temperatures (dynamic)^43^). Naturally, NNs trained on the coordinates (latitude and longitude) and day of year yield a decent fit, as they pick up the seasonal cycle which comprises the majority of the soil moisture signal. However, the SMAP SM is not reducible to a pure location-wise seasonal cycle (and, as will be shown later, is also not reducible to the seasonal cycle in brightness temperature) and in fact, deviations from this seasonal cycle are the most important part of the signal. When latitude-longitude data is added to the NN, the use of only one incidence angle data performs almost as well as using four incidence angles. As a result, single-angle data was chosen for the sake of model parsimony and considering the fact that adding other incidence angles would reduce the data size (due to missing data in other incidence angle data). The use of convolutional NN and NN trained on PCA rather than on raw TB data is tampered by the data availability and overall, does not provide any benefits in terms of performance metrics. Altogether, for the SMOS-SMAP pair, SMOS TB from one incidence angle 42.5° in two polarizations (H and V) and geographic coordinates (latitude and longitude) were taken as a NN input, and dense, deep NN was chosen as the best-performing NN architecture.

For AMSR-E and AMSR2, the data is available from five frequency channels in two polarizations each. For these sensors, there is a constant number of data observation points per frequency (there is no missing data issue such as in SMOS). Through a trial and error process of feature selection, a subset of 4 brightness temperatures–10.7 GHz in V-polarization, 18.7 GHz in H-polarization, 36.5 GHz in H-polarization and 89.0 GHz in V-polarization–accompanied by the latitude and longitude were chosen as a minimal subset resulting in high NN performance. AMSR-E/2 sensors do not collect information in L-band, associated most closely with soil moisture information. However, through NN training to match SMAP SM, we were able to achieve good SM retrievals based on this subset of TBs. A full description of NN inputs and outputs for all NN is given in Table 1.

**Table 1:** NN input-output configuration for NN_SMOS→SMAP_ and NN_AMSR→SMAP_, including NN_AMSR→SMAP_ transfer learning.

	Input	Output	Dates	Characteristics
NN_SMOS→SMAP_	Residual SMOS TB: - 42.5 H-polariz - 42.5 V-polariz Latitude Longitude	Residual SMAP SM	2015/03/31 - 2020/08/27	7 hidden layers, 1050 neurons in each
NN_AMSR→SMAP_	Residual AMSR2 TB: - 10.7 GHz V-polariz - 18.7 GHz H-polariz - 36.5 GHz H-polariz - 89.0 GHz V-polariz Latitude Longitude	Residual SMAP SM	2015/03/31 - 2020/08/27	7 hidden layers, 1050 neurons in each
NN_AMSR→SMAP_ transfer learning	Residual AMSR-E TB: - 10.7 GHz V-polariz - 18.7 GHz H-polariz - 36.5 GHz H-polariz - 89.0 GHz V-polariz Latitude Longitude	Residual NN_SMOS→SMAP_ output	2010/01/17 - 2011/10/03	First 3 layers non trainable

The full data available for training contains 79,310,130 data triples (TB-H, TB-V, SM) for SMOS-SMAP NN and 53,819,920 data quintuples (TB-H10, TBV18, TBH36, TBV89, SM) for AMSR-SMAP NN. From each of these datasets, 80% of the data was assigned to training, and 20% was assigned to testing. During the NN training, the training dataset was additionally divided such that 20% of it was used for validation. For each NN training, validation mean squared error *mse* was used to assess NN fit and to avoid overfitting. In particular, training *mse* and validation *mse* were plotted, we made sure to stop the training before validation *mse* starts to increase. Additionally, we checked that the assessment metrics on the test sample do not deteriorate significantly in comparison to the metrics obtained on the training part of the sample. NN hyperparameters for all NN were optimized using SHERPA^44^. The final NN configuration consists of 7 layers with 1050 neurons in each.

### Training scheme

Since our goal is to create a consistent dataset matching SMAP SM skill, but SMAP is not available over the entire period with different satellites, a special scheme was developed to train the NN on the available patched data. The illustration of the adapted scheme is given in Fig. 1 and the corresponding NN input-output is detailed in Table 1.

The subsequent NN training scheme includes **transfer learning**. Transfer learning is a special NN learning framework that is aimed at improving learning performance when a new training dataset is believed to have a different set of features or a different distribution in comparison to the initial training dataset^45^. Let us assume an NN was trained on a large dataset. Then, a new dataset is obtained that might be different from the original training dataset. However, it is usually not optimal to completely retrain or train a new NN. Instead, a part of the initially trained NN skill is preserved and the NN is additionally tuned to the new dataset. There are various approaches to transfer learning; in our case, we take the trained NN, force a part of the weights to stay as they are (i.e., are not trainable) and then the rest of the weights can be adjusted during the training on a new dataset. When performing transfer learning, it is important to find a balance between preserving past NN skill and tuning the NN to the new dataset. It is done by using fewer epochs in the training, and checking the NN performance during the testing phase: if training performance is rising while testing performance is dropping, the NN is overfitted to the new data and has lost its generalization quality.

Next, we will look at the full training scheme used to create our dataset.

- First, an initial NN was trained on (SMOS TBs–SMAP SM) pairs for the period when the two data sets overlap, namely, starting on March 31, 2015. This NN is labeled NN_SMOS- > SMAP_. NN inputs are SMOS TB residuals, latitude and longitude. NN targets are SMAP SM residuals.
- Then, NN_SMOS→SMAP_ can be used backward in time on the full SMOS TB dataset, creating an SM product covering 2010–2020. The NN_SMOS- > SMAP_ output is the residual SM, the full SM is then obtained by simply adding back the previously derived seasonal cycle SM at every grid point. Due to the seasonal cycle calculation approach, it is assumed that SM seasonal cycle is constant for a given location and does not change from year to year. All potential deviations, including trends, should be picked up through the residual component of SM.
- A second NN, labeled NN_AMSR→SMAP_ was trained on (AMSR2 TBs–SMAP SM) pairs for the period when the SMAP and AMSR2 datasets overlap (from March 31, 2015). NN inputs are AMSR2 TB residuals, latitude and longitude. NN targets are SMAP SM residuals. However, the cumulative distribution function (CDF) of the output of this NN is not necessarily identical to the output of NN_SMOS→SMAP_. Moreover, the data from AMSR-E and AMSR2 are also not identically distributed and exhibit a known bias between the two^46^.
- To match the outputs from NN_SMOS→SMAP_ and NN_AMSR→SMAP_, transfer learning was performed such that NN_AMSR→SMAP_ is additionally trained on the (AMSR-E TB–NN_SMOS→SMAP_ output SM) pair for the period when AMSR-E dataset and NN_SMOS→SMAP_ output overlap (2010/01/17–2011/10/03). In our transfer learning approach, the first 3 layers (out of a total of 7, see Table 1) were kept fixed while the weights in the layers upstream were adjusted. NN inputs are AMSR-E TB residuals, latitude and longitude. NN targets are CASM SM residuals.
- Then, the additionally trained NN_AMSR→SMAP_ can be used to retrieve SM residuals for the whole period that AMSR-E data is available, namely, starting in 2002. The full SM can be obtained by adding back the seasonal cycle SM.
- Finally, by concatenating the NN_SMOS→SMAP_ and NN_AMSR→SMAP_ outputs, a consistent SM data set was obtained covering the years 2002–2020. The mean between the two NN outputs is taken for the period when they overlap (2010/01/17 - 2011/10/03) as they are consistent in quality (see below Section CASM dataset, also Supplementary Fig. S7).

### Uncertainty

Characterizing the uncertainty of the results is an important part of building a reliable dataset. NN are by definition providing deterministic prediction and thus do not routinely include uncertainty quantification. While the dropout-based approach presented in^47^ (i.e. randomly removing connections within the NN) provides an easy and simple way to characterize NN-based structural uncertainty, it cannot be used in this case. Indeed, dropout is used to prevent overfitting, while the used NN architecture does not show any signs of overfitting (See Supplementary Fig. S4) but rather is built to be parsimonious (since training a NN of this scale on a very large remote sensing data sets is computationally expensive). For such NN configuration, adding dropout leads to inevitable degradation in performance, which is not desired. Instead, a different approach was adopted to characterize uncertainties. It comprises an explicit treatment for data input (aleatoric) and structural (epistemic) uncertainty through the following two steps.

1. Data input (aleatoric) uncertainty is considered through explicit input (TB) data sampling within a standard deviation of the **residual** part of the signal. In particular, a noise parameter with ${\mathcal{N}}(0,0.1\cdot std({\rm{residuals}}))$ was added to each TB, where TB residuals standard deviation *std*(residuals) was calculated individually for each grid point. This introduces a small noise in the input signal. As a reminder, the residuals are defined as the full SM signal minus the fitted seasonal cycle. Since the seasonal cycle is purposely defined to be consistent and invariant from year to year, the residuals include useful information – extreme events signature, potential trends,–but also, inevitably, noise. These residuals are not equivalent to noise in the signal, but also the actual errors of the full signal cannot be used as residuals’ errors due to the difference in the full signal vs. residuals amplitude. For that reason, the actual TB errors cannot be used directly in the noise analysis, and a random noise is added instead.
2. Structural (or epistemic) model uncertainty is considered through independent training of seven NN (with the same architecture and hyperparameters, so-called deep ensembles^48^). Retraining the same NN from a different random initiation (random initial weights) results in a random sample of trained network parameters. Initialization randomness and differences due to the stochastic gradient descent together represent the full model error^49^. Then, this sample allows a simple, Bayesian assessment of the NN structural uncertainty by taking the mean and deviation of the output across the sample.

For each of the independently trained NN (structural uncertainty), 10 forward runs were performed using the noisy input (input uncertainty) resulting in a total sample of 70 NN outputs. The final version of the product provides SM mean and range separately for the epistemic and aleatoric uncertainties since data uncertainty depends on the chosen level of the input noise.

## Data Records

A new consistent global 19-year soil moisture product is created using machine learning and different microwave remote sensing products that are merged optimally to create a long-term consistent product^28,29^. Its promising characteristics include spatial and temporal homogeneity, good interannual variability, and skill on the extremes (assessed as correlation, *R*^2^, and *mse* of the CASM residuals vs. SMAP residuals, where residuals are the full SM signal minus the fitted seasonal cycle). Additionally, aleatoric and epistemic uncertainty estimates are provided in order to give uncertainty quantification of the retrieval as a function of time and location. The introduced methodology shows an emergent characteristic of larger uncertainty for older retrievals as would be expected. The spatial distribution of the regions of reduced performance corresponds to the regions of lower skill for the remote sensing sensors such as in regions of high biomass or with very dry soils (e.g. deserts) where the variability is muted. In the future, the dataset can be updated and improved upon, as more SMAP, SMOS, and AMSR-E/2 data become available for the NN training. At the same time, new SMAP SM data can be directly added to the CASM dataset since the algorithm is designed to match SMAP SM data characteristics.

Our CASM SM dataset^28,29^ covers the period ranging from June 2002 to August 2020 with 3-day temporal resolution and spans globally (from −60 to 80 degrees latitude) at 25 km spatial resolution. The dataset is available to the public at 10.5281/zenodo.7072511^28^.

## Technical Validation

### CASM dataset

**Fig. 2:**
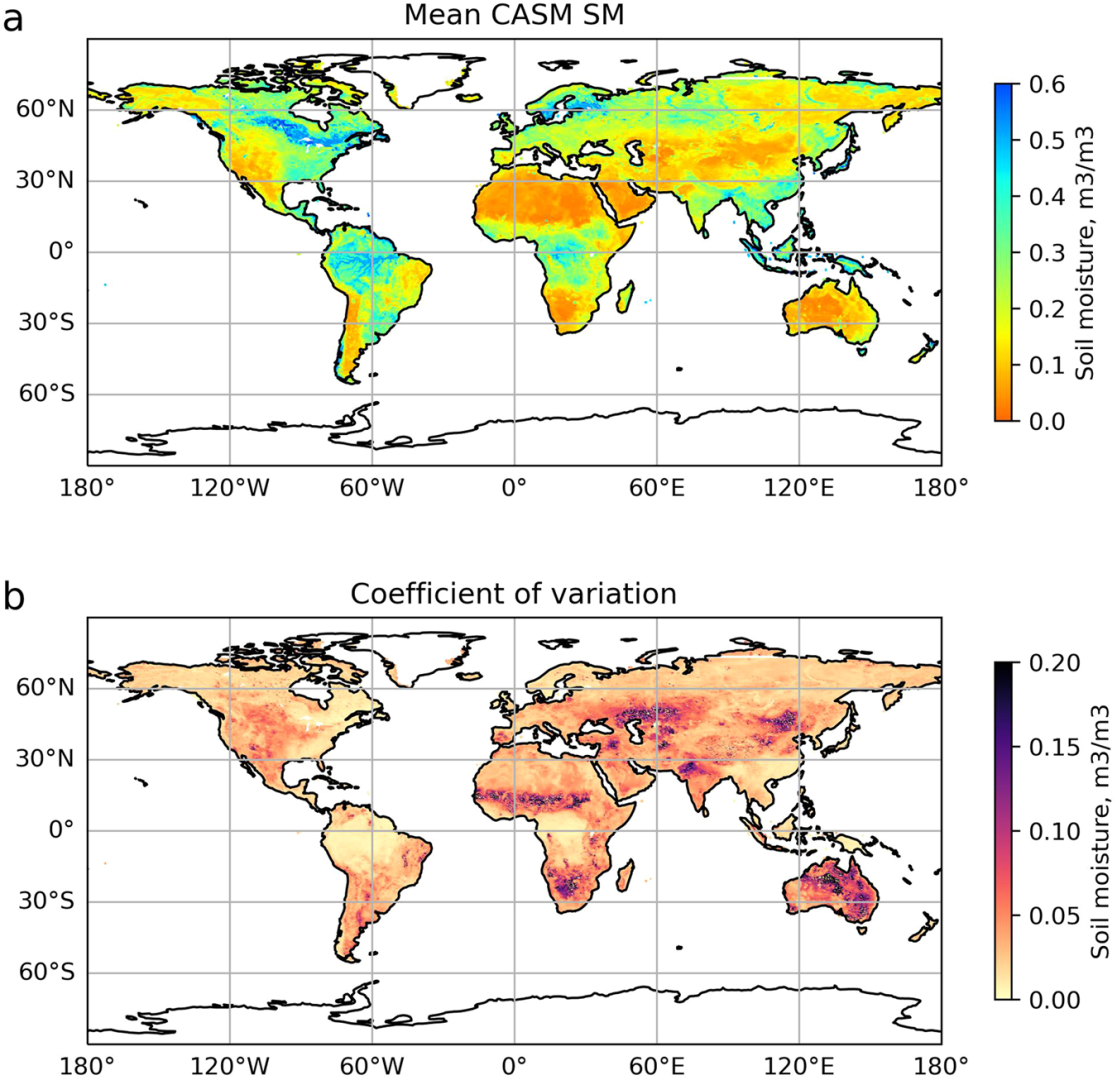
Global average soil moisture (**a**) and its coefficient of variation (**b**) from combined NN_SMOS→SMAP_ and NN_AMSR→SMAP_ outputs.

Global average CASM SM over 2002–2020 is presented in Fig. 2a. For comparison, the global average SMAP SM for the years 2015–2020 is shown Supplementary Fig. S5A. While local patterns of soil moisture may vary significantly, temporal averages over 2002–2020 and 2015–2020 should not have apparent visual discrepancies, since these maps represent climatology. The CASM SM dataset correctly identifies and accurately matches the global distribution of arid and moist regions to the ones of SMAP SM. The spatial correlation between temporally averaged CASM and SMAP SM for the overlapping years is shown in Supplementary Fig. S6A. Over most regions of the globe, the correlation between the two datasets is between 0.75 and 1, and the regions of low correlation correspond to the regions where remote sensing SM is less reliable due to technical characteristics of the microwave band retrievals, for example, tropical regions with dense vegetation, or highlands. It is more challenging to achieve a high correlation between the residuals, i.e. SMAP SM data minus seasonal cycle vs. CASM SM data minus seasonal cycle (Supplementary Fig. S6B). However, this variable is essential since it illustrates the model’s predictive skill on the extremes. Most of the globe is still characterized by high correlation (*R* > 0.75), however, the zones of lower correlation expand in the above-mentioned regions of the world, and additionally include the Northern Rocky Mountains, Himalayas, and highlands of Siberia, where SM standard deviation is low, and signal-to-noise ratio is low, which complicates meaningful NN retrievals.

Figure 2b shows the mean global coefficient of variation (CV) averaged over 2002–2020. The regions with higher CV (higher than 0.15 m^3^/m^3^) correspond to the arid and transitional regions between arid and wet climates (West and South Africa, Mid-West US, Central Asia, and most of Australia) where soil moisture variations are important. These regions correspond with regions of strong land-atmosphere coupling^50^. Since changes in soil moisture can be broadly described as a mass balance between input and output fluxes–precipitation, drainage, runoff, and evapotranspiration, dry and transitional regions are the regions where evapotranspiration is strongly coupled to and limited by soil moisture^2^ and hence, we expect to see there the most SM variability there. For the rest of the globe, the range of CV is between 0–0.05 m^3^/m^3^, with a global mean 0.02 m^3^/m^3^.

**Fig. 3:**
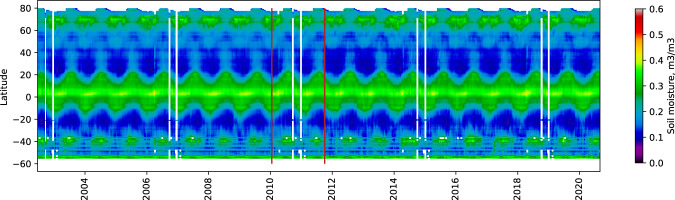
Hovmöller diagram showing the mean values of SM per latitude. NN_AMSR→SMAP_ and NN_SMOS→SMAP_ overlap is between two red vertical lines corresponding to 2010-01-17 and 2011-10-03.

The zonal average of the multi-sensor dataset is presented as Hovmöller diagrams in Fig. 3. Overall, the zonal average is consistent throughout the whole period with some interannual variability present. NN_AMSR→SMAP_ outputs are slightly less detailed than NN_SMOS→SMAP_ outputs due to natural limitations of the AMSR TB data. For future research, additional ML methods can be considered, such as generative adversarial networks (GAN) that can overcome these limitations.

**Fig. 4:**
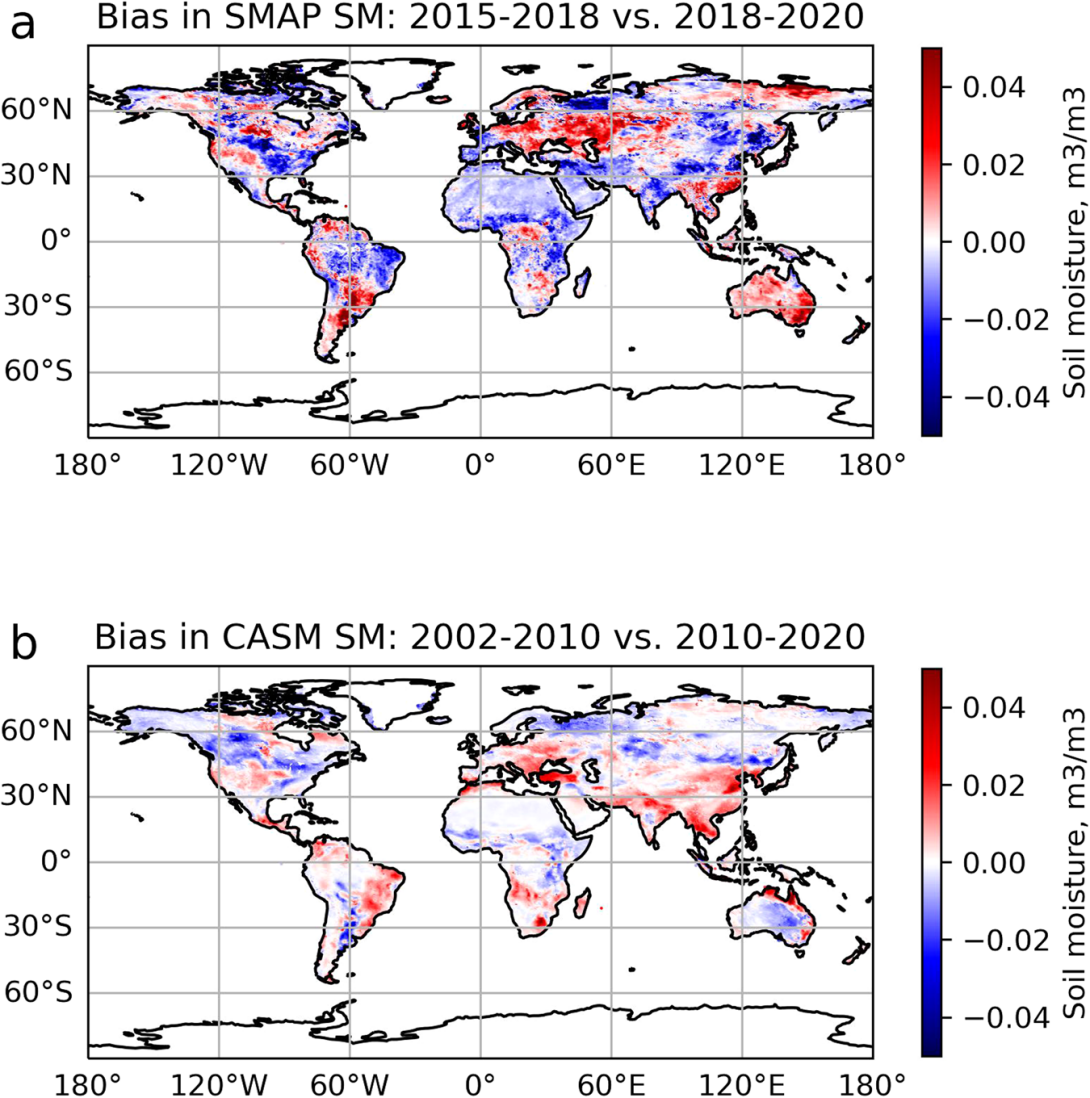
Spatial bias in SM (**a**) SMAP SM: the difference is between average SM 2015–2018 and average SM 2018–2020. (**b**) CASM SM: the difference is between average SM 2002–2010 and average SM 2010–2020.

Additionally, the NN_SMOS→SMAP_ and NN_AMSR→SMAP_ performances can be further explored by examining the output of these two NN and comparing the two outputs for the period when they overlap (01/17/2010 - 10/03/2011) (see Supplementary Fig. S7). We additionally examine a difference between NN_SMOS→SMAP_ and NN_AMSR→SMAP_ SM outputs and find that coefficient of variation is higher (*CV* > 0.15 m^3^/m^3^) for the NN_AMSR→SMAP_ in the same regions where higher CV was previously noted, and higher globally (with the global mean 0.05 m^3^/m^3^). To additionally investigate potential discrepancies between the two NN, we compare spatial biases between their outputs. Since some interannual biases can be present in the data and correspond to natural variability and SM response to external forcing (from natural, like ENSO, to anthropogenic, like change in irrigation practices, to climate change-related), we compare spatial biases in SMAP SM data between years 2015–2018 and 2018–2020 with spatial biases in CASM SM between 2002–2010 and 2010–2020 (Fig. 4). First of all, natural spatial biases in SMAP data (Fig. 4a) have larger amplitude than those of CASM data (Fig. 4, SMAP amplitude ±0.12 m^3^/m^3^, CASM amplitude ±0.04 m^3^/m^3^). That is most likely related to averaging effect since for SMAP, the average is calculated over 3 years, whereas for CASM, it is calculated over 9 years, which makes the signal smoother. Secondly, there is no evidence of consistent spatial bias between the two NN outputs as the regions of positive and negative differences for the two time periods roughly correspond to the regions also highlighted for the SMAP data (with allowances made for interannual variability). The high correlation (*R* = 0.98 for the full SM, and *R* = 0.80 for the residuals) and near 1-to-1 correspondence between the two NN outputs for the overlapping period (Supplementary Fig. S7) speaks in favor of the transfer learning scheme. The average performance of the NN_SMOS→SMAP_ and NN_AMSR→SMAP_ neural networks on training and test data is given in Table 2.

**Table 2:** NN performance metrics for NN_SMOS→SMAP_ and NN_AMSR→SMAP_, including NN_AMSR→SMAP_ transfer learning.

	Train / Residuals			Test / Residuals			Full / Residuals + seasonal cycle		
	R	R2	RMSE	R	R2	RMSE	R	R2	RMSE
NN_SMOS→SMAP_	0.73	0.49	0.03	0.70	0.49	0.03	0.97	0.94	0.03
NN_AMSR→SMAP_	0.78	0.60	0.028	0.78	0.60	0.028	0.97	0.93	0.028
NN_AMSR→SMAP_ Transfer learning	0.78 ± 2e-3	0.61 ± 3e-3	0.027	0.75 ± 1e-3	0.56 ± 3e-3	0.028	0.98	0.96	0.027

For all data without standard deviation reported, standard deviation was smaller than 10^−4^

### Advantages of the chosen seasonal cycle approach

On average, less than 1 percent of the locations did not have enough observations to calculate the seasonal cycle for the short (5-year) datasets (SMAP, 2015–2020) and less than 0.002% for the longer datasets (SMOS, 2010–2020, AMSR2, 2011–2020, AMSRE, 2002–2011). There were also only as few as 1–7 pixels (depending on a dataset) globally where the seasonal cycle could not be fitted to the observations. Overall, as expected, the seasonal cycle comprises the majority of the signal (Supplementary Fig. S8B in comparison to Supplementary Fig. S8A).

The NN approach showed a good ability to fit the residual anomaly signal (Supplementary Fig. S8C and E): The NN_SMOS→SMAP_ correlation *R* on this residual was 0.73 for the training data, and 0.70 for the test data, with *R*^2^ = 0.49 and root mean square error *RMSE* = 0.03 m^3^/m^3^. Correlation of the full SM (NN_SMOS→SMAP_ output residual + calculated seasonal cycle) to the original SMAP SM reached *R* = 0.97 (*R*^2^ = 0.94) further demonstrating the confounding effect of the seasonal cycle on the retrieval. Global mean bias (in comparison to SMAP for 2015–2020) is as low as −6.9⋅10^−5^ m^3^/m^3^ and does not have any apparent spatial patterns (Supplementary Fig. S5B).

To additionally check the quality of the chosen seasonal cycle treatment strategy (sine wave), an NN with the same NN hyperparameters was trained on the full TB data to match the full SM data (i.e. without seasonal cycle removal) and resulting in a correlation *R* = 0.95. The relative success of the NN trained on the full data (without deseasonalization), i.e. *R* = 0.95 vs. *R* = 0.97 on the deseasonalized data can be explained by the fact that the seasonal component comprises the majority of the signal. However, the true NN skill on the extremes is not clear from this metric. To elucidate that, the NN SM output from the “full signal” network was taken, the seasonal cycle was subtracted from the NN output, and these residuals were compared to the true SM residuals. The correlation between the residuals, in this case, was *R* = 0.55 and *R*^2^ = −0.02, indicating that while the seasonal cycle agnostic NN was able to capture some skill on the extremes, much of the performance comes from seasonal cycle matching while the extremes predictions are no better than noise (thus, the negative *R*^2^ on the residuals). Hence, while the NN trained on the full signal can achieve similar results if compared via correlation and RMSE metrics, its ability to capture extremes is strongly improved when the seasonal cycle is removed from the data. Discussion regarding NN performance in predicting time series with strong seasonality^51,52^ suggests that deseasonalizing data is an effective strategy to improve NN predictions. The improvement achieved for predictions of the extremes is very important for the further use of the created SM dataset. Indeed, in light of the recent increase in the frequency of extreme events like floods and droughts, examining their attribution and connection to different drivers, including SM, draws more attention (e.g.^53,54^). Attribution of the extreme events to extreme SM conditions will be impossible if an SM dataset represents the extreme SM values incorrectly.

### Comparison to *in-situ* observation

**Fig. 5:**
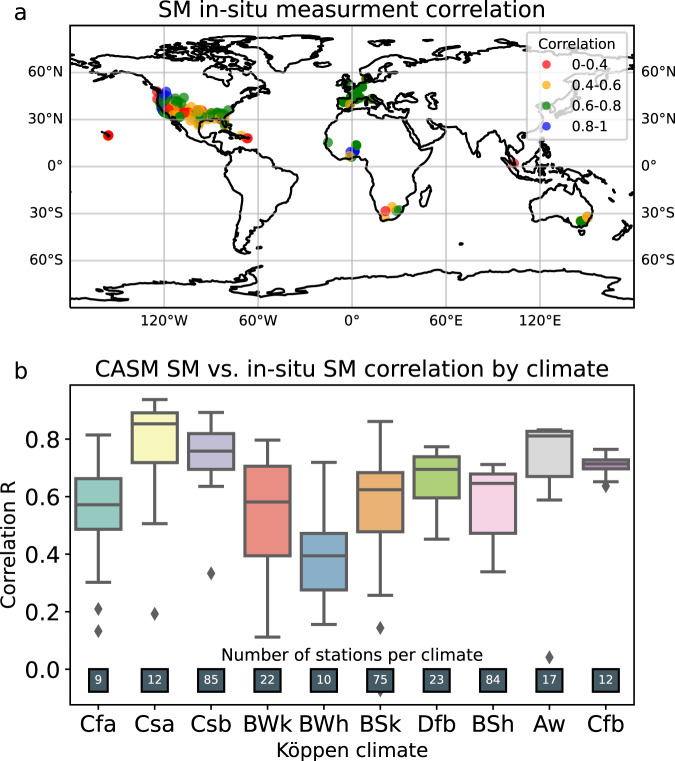
Correlation between ISMN SM and CASM SM (**a**) Global distribution of CASM vs. *in-situ* SM correlation (**b**) CASM vs. *in-situ* SM correlation per climate type.

Soil moisture from our CASM^28,29^ dataset was then compared to *in-situ* observations from the ISMN. Overall, 367 stations satisfied the criteria described in Section *In-situ* data. The majority of the stations satisfying those criteria are located in the United States and Europe and span across 16 climatic zones according to the Köppen climate classification. The correlation between the data measured at these stations and the SM from CASM dataset is depicted in Fig. 4. Unfortunately, the number of stations suitable for the comparison is not uniform across the globe, and hence, it is challenging to assess a particular spatial pattern that would emerge when comparing the two data sources. From the correlation categorized by climate (Fig. 5b), only climates with at least 9 stations per climate are shown), it seems that CASM SM dataset correspondence to *in-situ* measurements is low for hot desert (BWh). BWh is characterized by a very low SM (which is also indirectly confirmed by the lowest unbiased RMSE–Supplementary Fig. S9) that can cause reduced correlation. CASM also exhibits hindered performance in tropical climates most likely due to remote sensing products’ challenges in capturing SM signal under very dense tropical vegetation.

Overall, the total median correlation between station SM and CASM SM from the corresponding grid cell is 0.66 (mean 0.63) which is in the range of the existing SM products (0.47–0.78 according to^55^) and is very close to the corresponding correlation with the *in-situ* data of the SMAP SM dataset itself (median correlation between SMAP SM that was used as a target for this study–denoted SMAPL3E by^55^ is 0.65 when compared to 805 stations). No spatial aggregation was used to avoid introducing additional incoherence between this and the aforementioned study. Comparison to *in-situ* observations, while giving a certain measure of CASM dataset performance, is not free of issues. First and foremost, this comparison is a point estimation from an on-ground sensor compared to an estimation over a grid cell, which naturally introduces heterogeneity. Secondly, deteriorated performance could be a result of poor *in-situ* measurements, drifts in a sensor, or incorrect sensor calibration even when a good quality flag has been reported (e.g. Supplementary Fig. S10a). Finally, the retrieved data can be of bad quality due to remote sensing technology imperfections or the presented methodology errors. We studied a random sample of time series of SM measured *in situ* vs. CASM time series from the corresponding grid cell and found no indication of global bias or variability issues, despite the discrepancies between the time series at certain locations. The potential reasons for these discrepancies are beyond the scope of this paper. Local biases may exist, though are not large, according to Supplementary Fig. S5B, and will be a subject of the follow-up study. Naturally, since the CASM dataset is created to be consistent with the SMAP SM, in some cases, while CASM SM correlation with *in-situ* data is low, the same CASM SM correlation with SMAP SM is significantly higher (e.g. Supplementary Fig. S10 a, CASM-station correlation is 0.17, while CASM-SMAP correlation is 0.80). Finally, Supplementary Fig. S10b illustrates the difference between the seasonal cycle signal and the SM signal on a local level. Despite the minimal visual difference between the actual SM output and seasonal cycle on the global longitude-averaged scale (Supplementary Fig. S8A vs. Fig. S8B), the difference between the two is significant for the individual locations. Supplementary Fig. S10b also displays that the used seasonal cycle is not necessarily matched the climatological SM cycle by amplitude for a given location, but rather plays an auxiliary role to aid NN training.

### Uncertainty estimation

**Fig. 6:**
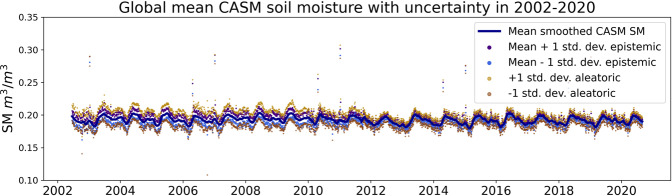
Temporally averaged structural (epistemic) and data (aleatoric) uncertainty averaged over the globe.

**Fig. 7:**
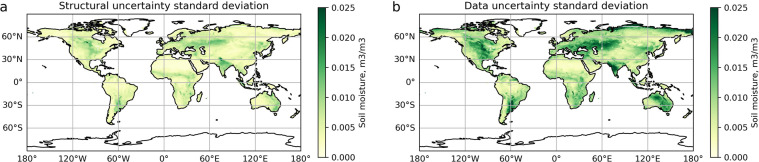
Spatially averaged standard deviation of (**a**) structural and (**b**) data uncertainty of retrieved SM.

Following the uncertainty estimation procedure, structural (epistemic) and data input (aleatoric) uncertainty was calculated. Temporally and spatially averaged uncertainties are shown in Fig. 6 (mean uncertainty) and Fig. 7 (standard deviation of uncertainty). Structural uncertainty is very small (the mean is ±0.005 m^3^/m^3^) and does not have notable spatial patterns (Fig. 7a). In fact, it is of the order of magnitude of the SMAP SM range (Supplementary Fig. S11). Hence, the introduced methodology does not increase SM retrievals uncertainty. Data uncertainty is introduced as a small (less than 10%) noise to the TB residuals and is aimed to illustrate the model’s resilience. Indeed, the NN outputs obtained from the noisy inputs are reasonably contained (the mean is ±0.009 m^3^/m^3^) and show no signs of instability. Spatial distribution of the uncertainty is the most pronounced for the cropland regions and tightly matches their spatial distribution around the globe–in India and South Asia, Western and Central Europe, Central America, sub-Sahara and East Africa, South America and East Australia (Fig. 6b). For practical use, we recommend referencing structural (epistemic) uncertainty, since aleatoric uncertainty presented here depends on the arbitrarily chosen level of noise applied to the input data. In general, the chosen level of noise should depend on the actual uncertainty of the input data. However, TB uncertainties, if reported, are given in relation to the full TB signal, and the way to propagate it through seasonal cycle-residual decomposition is not straightforward. Increased uncertainty for the 2002–2010 part of the dataset (the mean structural uncertainty in 2010–2020 is ±0.003 m^3^/m^3^, the mean structural uncertainty in 2002–2010 is ±0.007 m^3^/m^3^) is a reflection of the higher frequency and higher uncertainty of older satellite platforms; it is therefore expected and, to some extent, desirable characteristic. Interestingly, however, this characteristic is an emergent property of the multi-staged training scheme (as in all other respects, NN_AMSR→SMAP_ and NN_SMOS→SMAP_ are treated equivalently, and the introduced input noise level is the same).

### Comparison to other long-term soil moisture datasets

The goal of this study is to create an SM dataset of consistent quality. SMAP SM (in particular SMAPL3E^55^) is chosen as a target SM. The resulting dataset inherits all shortcomings of the target SMAP SM, however, if the dataset is consistent in time, the goal of this study is achieved.

Since each sensor has a specific set of characteristics that differ from other sensors, such as the observed variable (scattering coefficient, brightness temperature), the wavelength band (X, C, and L-band) and corresponding penetration depth, the polarization, the incidence angle, the spatial resolution, the retrieval algorithm, surface roughness or radio frequency interference (RFI)^31,56,57^, the resulting inferred soil moisture dataset from different sensors are likely going to have inconsistent distributions and will require rescaling and recalibration, pixel-wise, prior to merging. Merging various sensor retrievals *a posteriori* requires major assumptions on nonlinear, state-dependent, and thus geographically varying relationship between those sensors and on their distribution. Another approach employs local CDF matching of the observations to a reference CDF, e.g., from a reference retrieval or model. However, if processes (such as irrigation) are missing in the model and are present in the remote observation, such rescaling can lead to the omission of the corresponding physical process^58^. An NN has the advantage of being both nonlinear, and state-dependent, and thus naturally imposing a global CDF matching (as it tries to match the retrieved product with the target, globally), as opposed to local CDF matching discussed earlier. An NN creates a data set that is directly consistent with the target data (either an SM model or an SM retrieval from other sources) and does not need any *a posteriori* distribution or bias correction, as it is directly handled by the neural network. The resulting data can be utilized for data assimilation studies without information losses. In that respect, we find our approach to be the most suitable to constructing a long-term dataset of consistent quality.

A simple but critical test of dataset consistency is the presence of artificial trends in the dataset, which would demonstrate that the dataset cannot be used for long-term soil moisture investigations (trend and variability). To this goal, we investigate the globally-averaged time series of several datasets. Supplementary Fig. S12 illustrates our approach by comparing three SM products available in 2002–2020: ESA CCI SM (v.07.1), Yao *et al*. SM product^59^, and CASM SM^28,29^ (this study). As can be seen from Supplementary Fig. S12A, ESA CCI SM is not consistent over 2002–2020, with biases not only in the mean but also in variance. Another example of an inconsistent SM dataset is shown in Supplementary Fig. S12B. Despite excellent performances on various metrics^59^, the dataset exhibits a clear shift in the data mean in 2002–2011 vs. 2012–2020. For comparison, CASM SM globally averaged time series are shown in Supplementary Fig. S12C. CASM SM shows much-improved stability over time, which is the primary purpose of our dataset. A more detailed analysis of the CASM dataset’s local and global performance is in the scope of the follow-up study.

## Usage Notes

The dataset is open to public use without limitation. The permanent storage is at 10.5281/zenodo.7072511, the data is stored as 19 separate yearly data files in NetCDF format. Additionally, the CASM dataset is openly available on Pangeo https://pangeo-forge.org/dashboard/feedstock/85, which provides easy access and cloud computation services.

Each data file contains coordinates and date, with the corresponding variables:

- **CASM soil moisture** is the soil moisture in the top soil level, in m3/m3.
- **Seasonal cycle** is the calculated auxiliary variable, in m3/m3.
- **Structural uncertainty** is epistemic uncertainty, in m3/m3.
- **Data uncertainty** example aleatoric uncertainty for a small (<10%) perturbation in the input data, in m3/m3.

## Supplementary information

Supplementary information

## Data Availability

All code is written in Python, the analysis is conducted using Columbia University high performance computing clusters (Ginsburg), and is available at https://github.com/os2328/CASM-dataset.
