# Supplementary material for: A Long-term Consistent Artificial Intelligence and Remote Sensing-based Soil Moisture Dataset
Source: Sci Data. 2023 Mar 22;10:154. doi: 10.1038/s41597-023-02053-x (PMC10033968; doi:10.1038/s41597-023-02053-x)
Supplement: Supplementary file 1 — Supplementary information [file 41597_2023_2053_MOESM1_ESM.pdf]

## Supplementary Information

This is supplemental material to the manuscript **"A Long-term Consistent Artificial Intelligence and Remote Sensing-based Soil Moisture Dataset"** by Olya Skulovich and Pierre Gentine.

Columbia University, Earth and Environmental Engineering Department, New York, NY 10027, USA

Corresponding author: Olya Skulovich (os2328@columbia.edu)

| **Network** | **Reference(s) or website** |
|---|---|
| AMMA-CATCH | http://www.amma-catch.org |
| ARM | https://www.arm.gov |
| DAHRA | Tagesson et al. (2015)[1] |
| FLUXNET-AMERIFLUX | https://ameriflux.lbl.gov |
| FR-Auqi | Al-Yaari et al. (2018)[2], Wigneron et al (2018)[3] |
| HOBE | Kang et al. (2014)[4], Jin et al. (2014)[5] |
| HYDROL-NET Perugia | Morbidelli et al. (2014)[6] |
| MySMNet | Kang et al. (2016)[7] |
| ORACLE | https://gisoracle.inrae.fr |
| OZNET | Smith et al. (2012)[8] |
| PBO-H2O | https://cires1.colorado.edu/portal/ |
| REMEDHUS | https://campus.usal.es/$\sim$hidrus/ |
| SASMAS | http://campus.usal.es/$\sim$hidrus/ |
| SCAN | http://www.wcc.nrcs.usda.gov |
| SMOSMANIA | Calvet et al. (2007)[9], Albergel et al. (2008)[10] |
| SOILSCAPE | Moghaddam et al. (2010, 2016)[11,12] |
| TAHMO | https://tahmo.org |
| TERENO | Zacharias et al. (2011)[13] |
| USCRN | Bell et al. (2013)[14] |
| USDA-ARS | https://www.ars.usda.gov |
| VAS | Lopez-Baeza et al. (2008)[15] |
| WSMN | Petropoulos and McCalmont (2017)[16] |

**Table S1.** In situ soil moisture data measurement networks

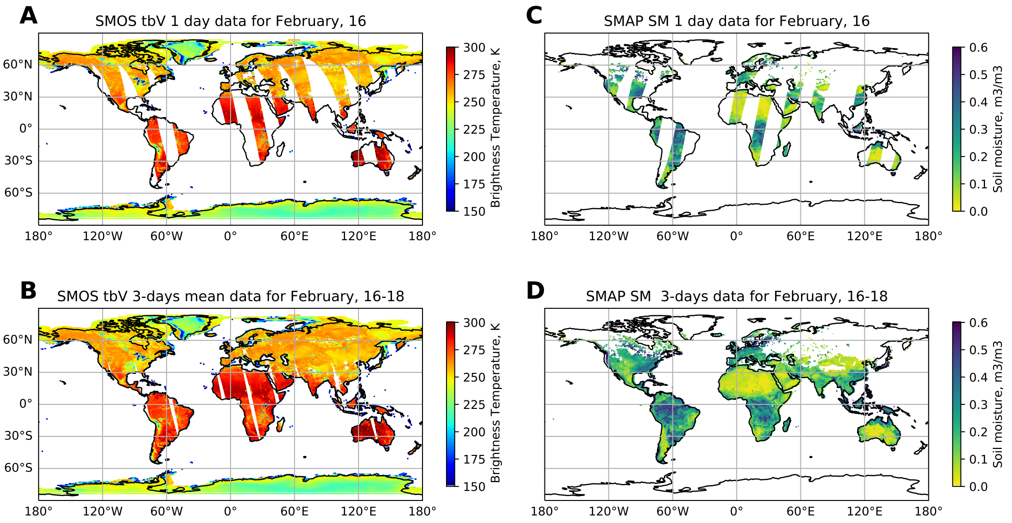


**Figure S1.** (A) Daily data collected by SMOS; (B) Daily data collected by SMAP; (C) 3-days mean by SMOS; (D) 3-days mean by SMAP

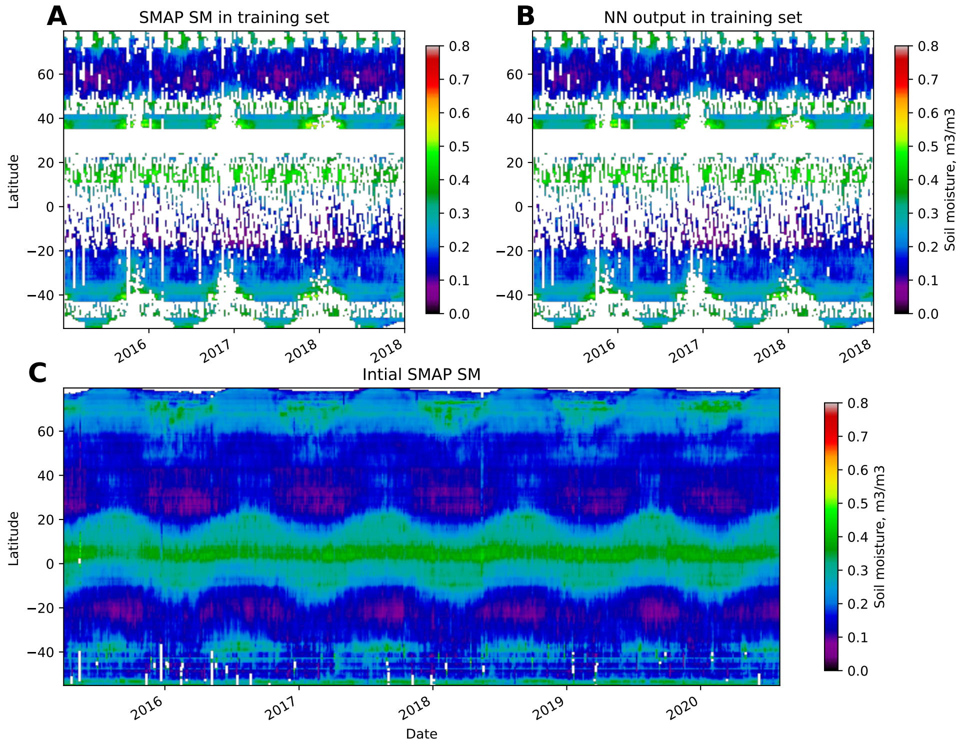


**Figure S2.** Hovmöller diagrams showing the daily mean values of SM per latitude for (A) SMAP data in the training dataset only for the regions that have SMOS data during the same day; (B) NN output SM for a NN trained on such training data set; (C) Original SMAP data

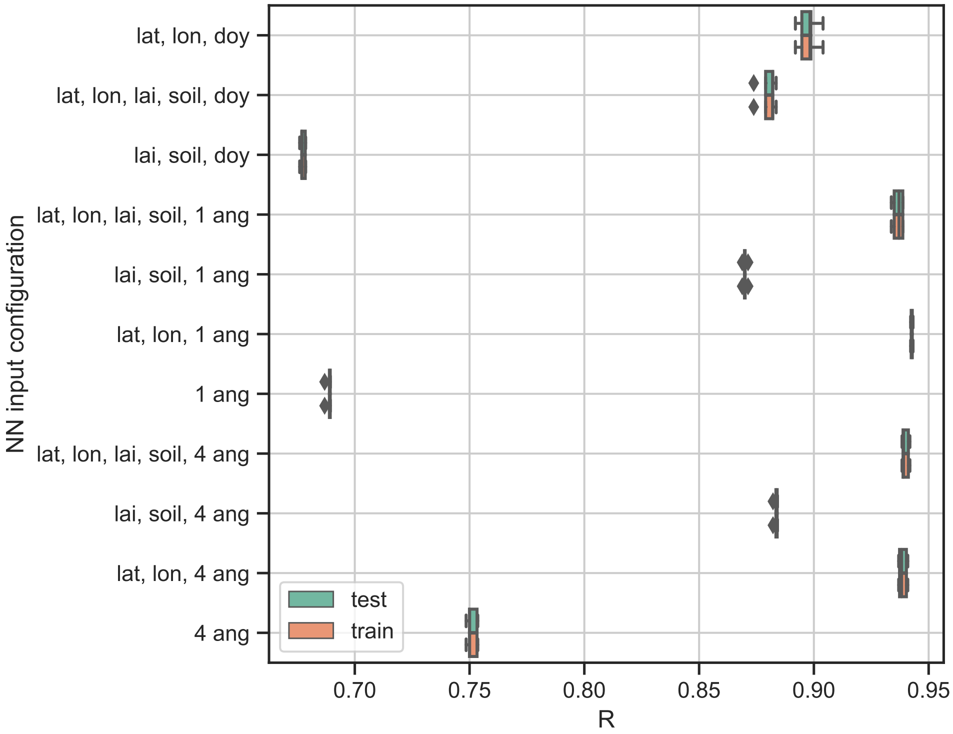


**Figure S3.** Train and test performance for a dense NN measured as correlation R between SMAP SM and NN output SM for a given input configuration, where *lat* is latitude, *lon* is longitude, *doy* is day of year, *lai* is LAI, *soil* is soil type, *1 ang* is SMOS TB from one incidence angle 42.5° (smallest number of missing data) in two polarizations, *4 ang* is latitude, SMOS TB from four incidence angles 37.5-47.5°in two polarizations.

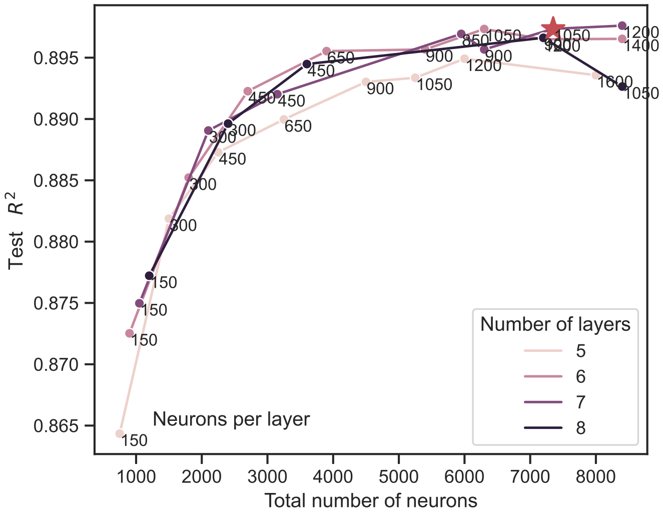


**Figure S4.** Test performance for NN with a different number of layers and neurons per layer. The total number of neurons in a NN is shown at X-axis while the number of neurons per layer annotates the curves. Correlation between the NN output and the true SM value on the test sample measured as $R^2$ is used as a measure of NN performance. The NN configuration used in this work (7 layers, 1050 neurons in each) is shown as a red star.

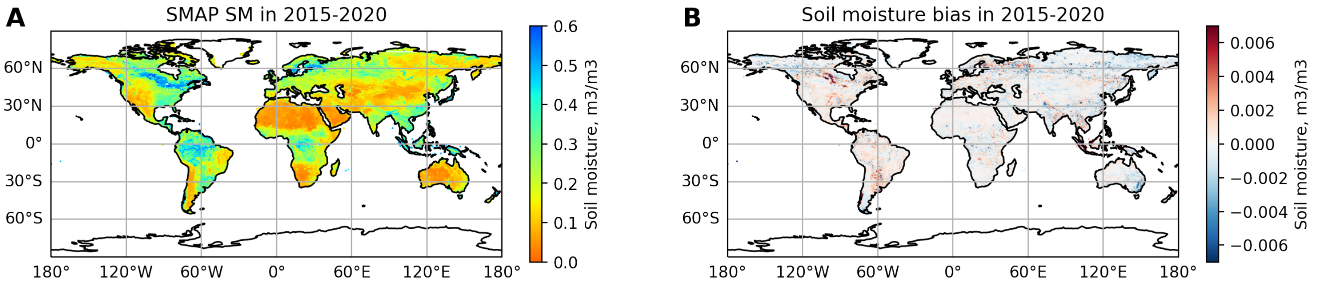


**Figure S5.** (A) Global average soil moisture from SMAP (B) Spatial distribution of a bias between CASM and SMAP SM for the period when the two datasets overlap (2015-2020)

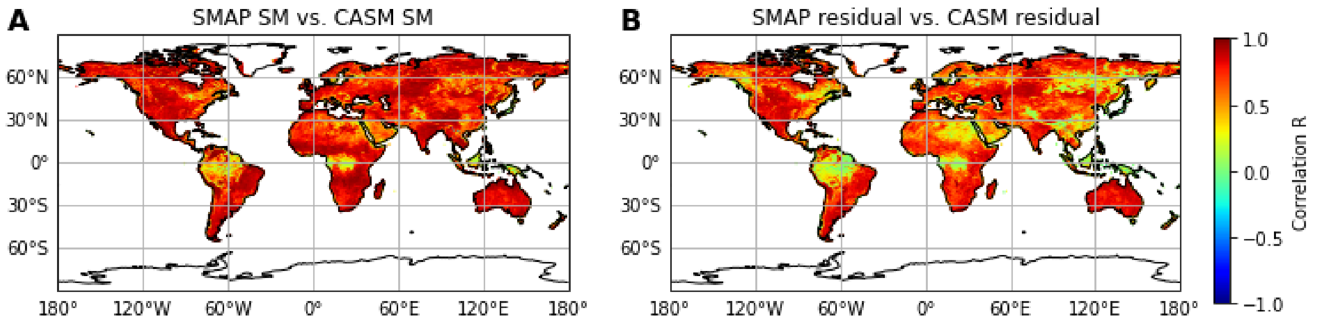


**Figure S6.** Correlation between SMAP and CASM for the full SM signal (A) and residual (B).

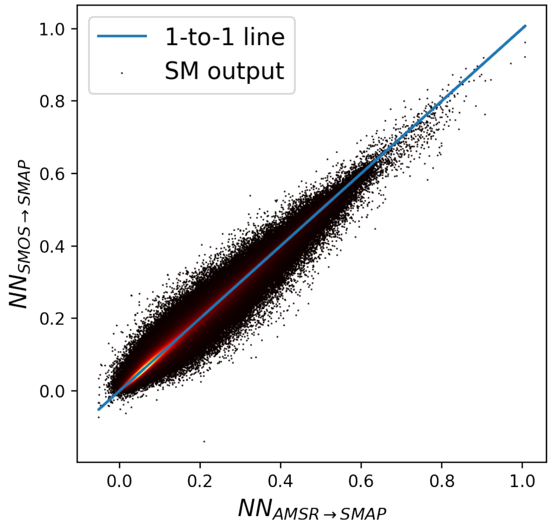


**Figure S7.** Kernel density estimation of $\mathrm{NN}_{\mathrm{AMSR}\rightarrow\mathrm{SMAP}}$ and $\mathrm{NN}_{\mathrm{SMOS}\rightarrow\mathrm{SMAP}}$ SM output for the period when the two overlap (01/17/2010 - 10/03/2011). A random sample of 420,000 points (out of 21.2 million).

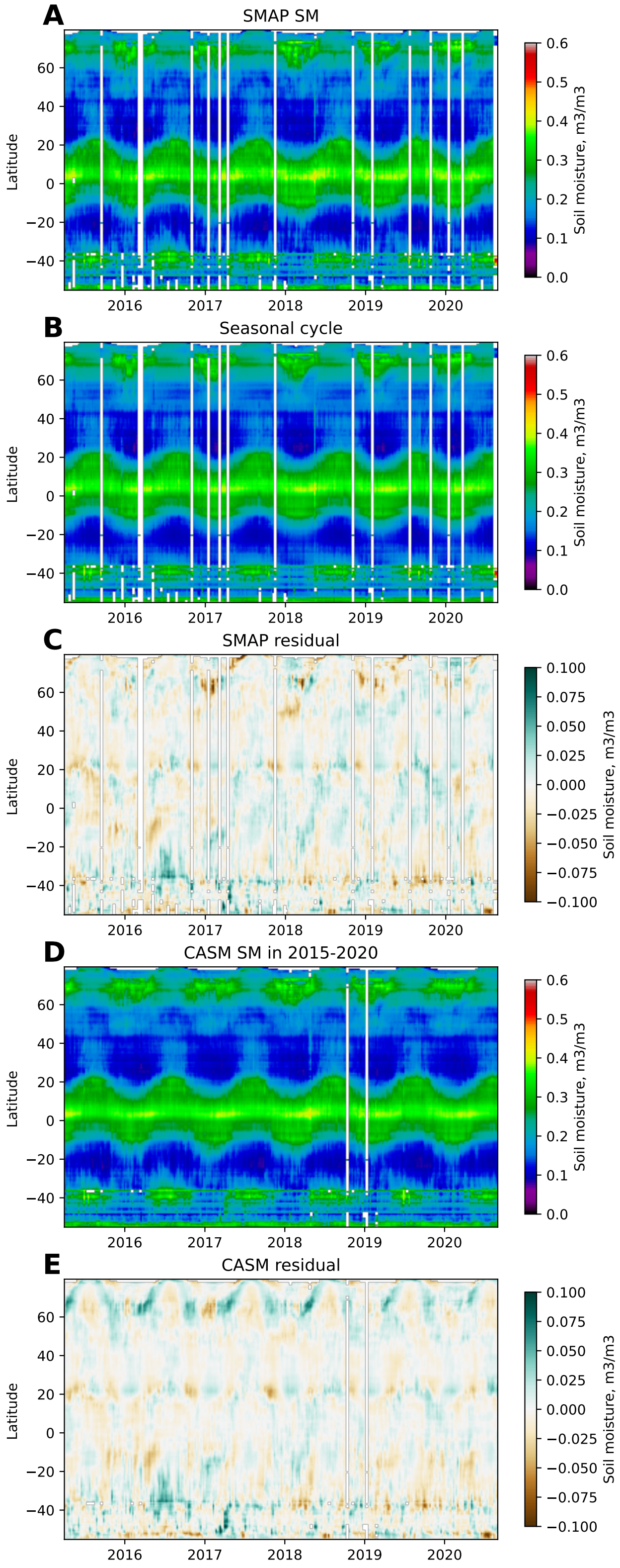


**Figure S8.** SM signal in 2015-2020. (A) Original SMAP SM. (B) Seasonal cycle. (C) Original residual SM. It is constructed as SMAP SM minus the seasonal cycle and used for $NN_{SMOS \rightarrow SMAP}$ training.(D) CASM SM constructed as the seasonal cycle plus $NN_{SMOS \rightarrow SMAP}$ residual output. (E) $NN_{SMOS \rightarrow SMAP}$ output SM residual.

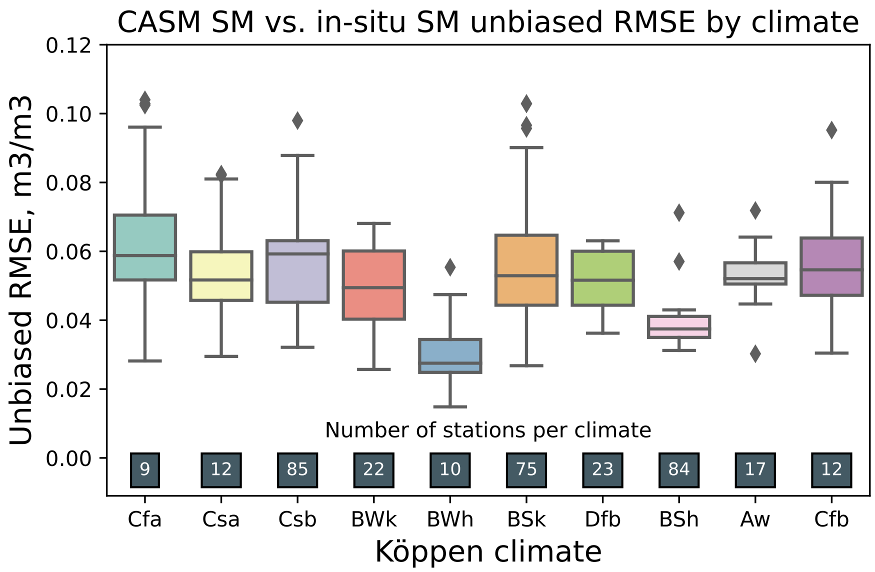


**Figure S9.** Unbiased RMSE between CASM SM and in-situ SM per climate.

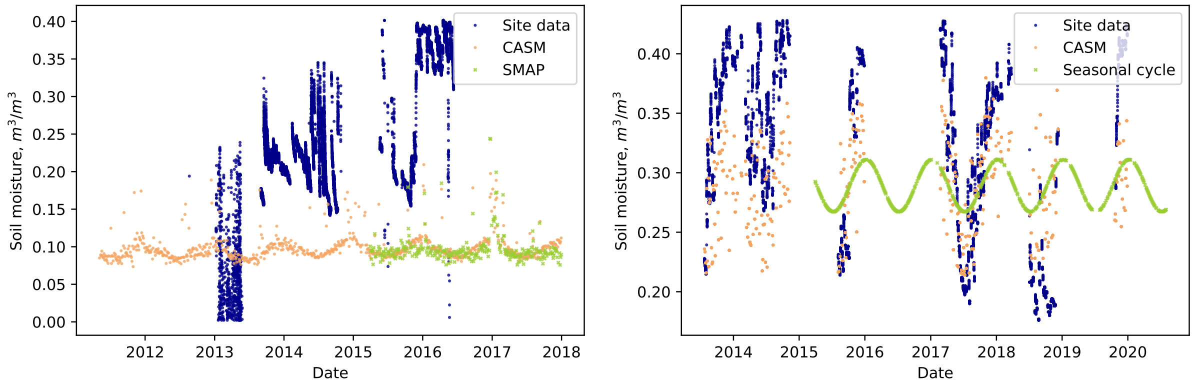


**(a)** Node119 (SOILSCAPE), AZ, USA

**(b)** Hoal-08 (HOAL), Austria

**Figure S10.** Example time series of soil moisture comparing CASM dataset and on-ground measurements. Naming convention: Station name (Network), State, Country

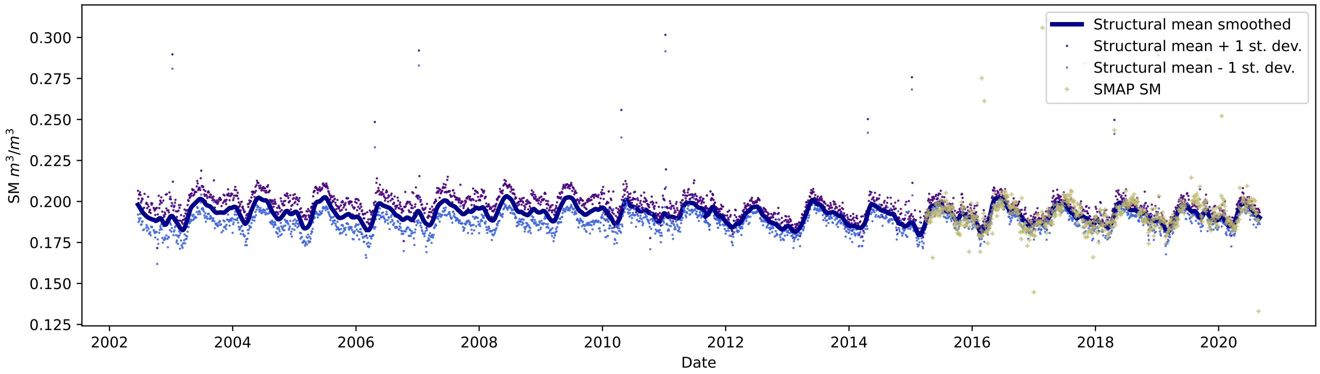


**Figure S11.** Temporally averaged structural (epistemic) uncertainty compared to temporally averaged SMAP SM range

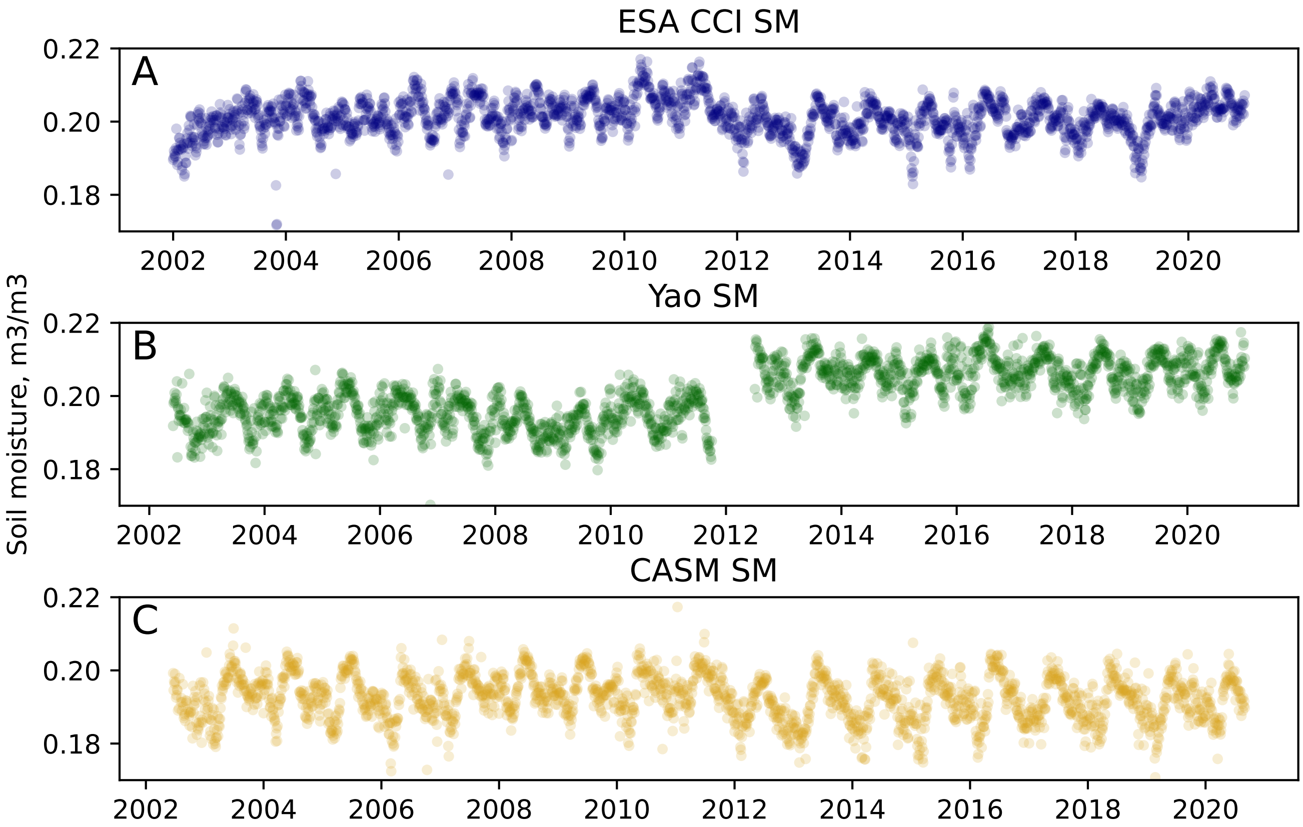


**Figure S12.** Globally-averaged time series of (A) ESA CCI SM, (B) Yao et al. SM (C) CASM SM. All datasets are first averaged to a 3-day mean and then globally.

## References


1. Tagesson, T. *et al.* Ecosystem properties of semiarid savanna grassland in West Africa and its relationship with environmental variability. *Glob. change biology* **21**, 250–264 (2015).
2. Al-Yaari, A. *et al.* The AQUI soil moisture network for satellite microwave remote sensing validation in South-Western France. *Remote. Sens.* **10**, 1839 (2018).
3. Wigneron, J.-P. *et al.* The aqui network: Soil moisture sites in the "les landes" forest and graves vineyards (Bordeaux Aquitaine region, France). In *IGARSS 2018-2018 IEEE International Geoscience and Remote Sensing Symposium*, 3739–3742 (IEEE, 2018).
4. Kang, J. *et al.* Hybrid optimal design of the eco-hydrological wireless sensor network in the middle reach of the Heihe River Basin, China. *Sensors* **14**, 19095–19114 (2014).
5. Jin, R. *et al.* A nested ecohydrological wireless sensor network for capturing the surface heterogeneity in the midstream areas of the Heihe River Basin, China. *IEEE Geosci. Remote. Sens. Lett.* **11**, 2015–2019 (2014).
6. Morbidelli, R., Saltalippi, C., Flammini, A., Rossi, E. & Corradini, C. Soil water content vertical profiles under natural conditions: Matching of experiments and simulations by a conceptual model. *Hydrol. Process.* **28**, 4732–4742 (2014).
7. Kang, C. S., Kanniah, K. D., Kerr, Y. H. & Cracknell, A. P. Analysis of in-situ soil moisture data and validation of SMOS soil moisture products at selected agricultural sites over a tropical region. *Int. J. Remote. Sens.* **37**, 3636–3654 (2016).
8. Smith, A. B. *et al.* The Murrumbidgee soil moisture monitoring network data set. *Water Resour. Res.* **48** (2012).
9. Calvet, J.-C. *et al.* In situ soil moisture observations for the CAL/VAL of SMOS: The SMOSMANIA network. In *2007 IEEE International Geoscience and Remote Sensing Symposium*, 1196–1199 (IEEE, 2007).
10. Albergel, C. *et al.* From near-surface to root-zone soil moisture using an exponential filter: an assessment of the method based on in-situ observations and model simulations. *Hydrol. Earth Syst. Sci.* **12**, 1323–1337 (2008).
11. Moghaddam, M. *et al.* A wireless soil moisture smart sensor web using physics-based optimal control: Concept and initial demonstrations. *IEEE J. Sel. Top. Appl. Earth Obs. Remote. Sens.* **3**, 522–535 (2010).
12. Moghaddam, M. *et al.* Soil moisture profiles and temperature data from SoilSCAPE Sites, USA, ORNL DAAC, Oak Ridge, Tennessee, USA (2016).
13. Zacharias, S. *et al.* A network of terrestrial environmental observatories in Germany. *Vadose zone journal* **10**, 955–973 (2011).
14. Bell, J. E. *et al.* US climate reference network soil moisture and temperature observations. *J. Hydrometeorol.* **14**, 977–988 (2013).
15. Lopez-Baeza, E. *et al.* Validation activities preparation for SMOS (soil moisture and ocean salinity) land products at the Valencia Anchor Station. In *EUMETSAT Meteorological Satellite Conference*, 8–p (2008).
16. Petropoulos, G. P. & McCalmont, J. P. An operational in situ soil moisture & soil temperature monitoring network for West Wales, UK: The WSMN network. *Sensors* **17**, 1481 (2017).
